# Endomicroscopy and Cancer: A New Approach to the Visualization of Neoangiogenesis

**DOI:** 10.1155/2012/537170

**Published:** 2012-01-12

**Authors:** Renato Cannizzaro, Maurizio Mongiat, Vincenzo Canzonieri, Mara Fornasarig, Stefania Maiero, Valli De Re, Federico Todaro, Paolo De Paoli, Paola Spessotto

**Affiliations:** ^1^Department of Gastroenterology, National Cancer Institute, IRCCS, Via F. Gallini 2, 33081 Aviano, Italy; ^2^Department of Experimental Oncology, National Cancer Institute, IRCCS, Via F. Gallini 2, 33081 Aviano, Italy; ^3^Department of Pathology, National Cancer Institute, IRCCS, Via F. Gallini 2, 33081 Aviano, Italy; ^4^Department of Clinical and Experimental Pharmacology, National Cancer Institute, IRCCS, Via F. Gallini 2, 33081 Aviano, Italy; ^5^National Cancer Institute, IRCCS, Via F. Gallini 2, 33081 Aviano, Italy

## Abstract

Probe-based Confocal Laser Endomicroscopy (pCLE) is a novel imaging technique for gastrointestinal endoscopy providing *in vivo* microscopy at subcellular resolution. It offers the possibility to analyze neoangiogenesis and vessel density *in vivo*. Angiogenetic switch is essential in cancer progression. Aim of the paper was to review the use of this imaging tool to analyze colorectal and gastric cancers vascularization *in vivo*. The aim is to provide the possibility of combining diagnostic evidences with vascularization and molecular profile to evaluate the efficacy of an antiangiogenic treatment in association with conventional therapy. pCLE can be considered a revolutionary method for real-time assessment of changes in vascularization pattern in this tumors and it may open the possibility to address the use of anti-angiogenic therapy in order to improve the outcome of the treatment.

## 1. Introduction

In recent years, endoscopic image quality has been highly improved thanks to very technologically advanced devices. One of these new gastrointestinal endoscopy methods is the confocal laser endomicroscopy (CLE) which has allowed for a confocal scanning microscope to be integrated into a conventional flexible endoscope. Potential applications include detection of neoplasia with targeting of biopsies, and the detection of inflammatory bowel disease, celiac sprue, and microscopic colitis [1]. CLE provides *in vivo* microscopy at subcellular resolution: imaging of the mucosal layer at resolution at approximately 1 micron and visualization of the cellular and subcellular structures as well as capillaries and single red blood cells are peculiar characteristics of this novel gastrointestinal endoscopy method. We experienced with the probe-based confocal endomicroscopy system (pCLE, Cellvizio, Mauna Kea Technology, Paris, France), in combination with videomosaicing which allows the reconstitution of panoramas of the tissues with the alignment of the input frames. We aimed therefore to use this new promising imaging tool to analyze the angiogenic process in colo-rectal and gastric cancer patients. This method discloses the possibility of a translational approach combining the confocal imaging not only with the diagnosis *in vivo* and the specific molecular profile of the patient, but also with the targeted antiangiogenic treatment.

## 2. Endomicroscopy and Tumors

The potential role of CLE has been explored in different pathologic conditions of the gastrointestinal (GI) tract, the possibility of diagnosing premalignant and malignant lesions of the GI tract being particularly important considering the prognostic implications. GI cancers represent a major cause of morbidity and mortality, with incomplete response to chemotherapy and poor prognosis in the advanced stages of the disease. Recently, CLE has been successfully applied in studies dedicated in particular to neoplastic Barrett's esophagus, and gastric and colorectal neoplasia. Since accurate diagnosis and staging are essential for therapeutic planning, CLE holds the potential for a strong impact in the screening and/or surveillance of GI tumors [2, 3]. CLE has been used in a pilot study also for detection of biliary malignancy [4]. All the studies performed revealed the clinical usefulness and predictive power for the high-resolution probe-based CLE for *in vivo* diagnosis of GI neoplasia and related precursor lesions during colonscopy. Based on characteristic morphological changes or due to characteristic single cells like globet cells in Barrett's esophagus, the promising technology of CLE enables already *in vivo* diagnosis of pathological mucosal conditions. However, confocal imaging holds the potential to go far beyond: the possibility to analyze the morphology and density of the blood vessels present on the surface of the tumors could also provide vital information for a more appropriate diagnosis and for a putative employment anti-angiogenic drugs during the treatment.

## 3. Angiogenesis Markers

The development of new blood vessels from the preexisting vasculature (angiogenesis) is an indispensable event both in normal and pathological conditions, such as cancer growth and development. Tumors will not grow beyond 1-2 mm unless the angiogenic switch is turned on [5], thus the formation of novel blood vessels is regarded as one of the most important events occurring in the neoplastic process [6]. In fact, the development of new vessels supplies the growing tumor with nutrients and oxygen, disposing metabolites and releasing growth factors that promote tumor cell proliferation [7]. Indeed tumors promote angiogenesis by secreting growth factors such as vascular endothelial growth factor (VEGF), hepatocytes growth factor, and platelet-derived growth factor that stimulate endothelial migration and proliferation [7–9]. The binding of VEGF to VEGFR triggers an intracellular signaling that is mainly mediated by MAPK and PI3K/Akt/mTOR pathways. This results in the expression of HIF-1a and induction of PDGF, FGF, G-CSF, TGF*β*, and angiopoietins, thus enhancing angiogenesis [10]. Angiogenesis is also indispensable to the metastatic process by providing large numbers of leaking blood vessels for vascular invasion [7]. To early detect and assess the extent of the intratumoral angiogenesis is thus crucial for a personalized and prompt antiangiogenic therapy. Besides the commonly used panendothelial markers such as CD31, CD34, Factor VIII, endoglin (CD105) has been proposed as a marker of tumor angiogenesis since the endoglin antibody binds preferentially to the activated endothelial cells that participate neovascularization [11, 12]. Endoglin is a receptor for the TGF-*β*1 molecule and was indeed found to be upregulated during neoangiogenesis [13]. Among the molecules specifically located along the blood vessels, MULTIMERIN2 (a.k.a. EndoGlyx-1) can be considered a good marker for the blood vessels. MULTIMERIN2 (MMRN2) belongs to the (EMI Domain ENdowed) EDEN protein family [14]. The protein was discovered during the search of novel markers of the vascular endothelium [15]. MMRN2 is in association with a high-molecular weight glycoprotein complex. Two subunits of this complex match the MMRN2 sequence and are likely the result of to posttranslational modifications. The other two subunits have not yet been identified [16]. MMRN2 can be considered a panendothelial marker being expressed both in normal and tumoral vasculature including hot spots of neovascularisation in some tumors [15–18]. The molecule was shown to be specifically deposited along the blood vessels in tight juxtaposition with endothelial cells and to be also present in the luminal side of the vessels [16]. Prior to our studies, its function though has remained obscure. Recent data collected in our laboratory [19] indicate that this molecule plays an important role in the regulation of endothelial cell function, tumor angiogenesis, and vessel homeostasis. To carry out these studies, we have developed both antihuman and antimouse antibodies against this molecule which were found to detect blood vessels in many tissue and tumor sections as well as the anti-CD31 antibody [19]. The use of this antibody was found to be suitable for the detection of blood vessels both in colorectal and gastric cancer sections as indicated by our results. Interestingly, MMRN2 was also demonstrated to play an important role in vessels maturation (in terms of pericyte coverage) and to regulate also endothelial cell permeability [19]. Thus this molecule may not only be a mere marker of blood vessels but also be important, depending on its expression, in predicting the vessel functionality. Indeed, the combination of the MMRN2 staining with the pCLE analysis could provide a more reliable evaluation of the “angiogenetic status” of the patients.

## 4. Endomicroscopy and Angiogenesis

High-resolution confocal imaging is achieved by using an exogenous fluorescence technique. Fluorescein is intravenously administered for specific *in vivo* imaging of human colorectal neoplasia and its use also allows the analysis of the vascular structure, morphology (irregular vessels) and leakiness (fluorescein outflow). Studies are currently underway to apply this new imaging tool for objective evaluation of the microvessel density in different stages of the neoplastic development and in conjunction with antiangiogenetic therapy. Preliminary data on the microvessel density for biliary cancers at the liver hilum [20, 21], for Barrett's esophagus [22], and for GI tumors [23, 24], are currently available.

**Figure 1:**
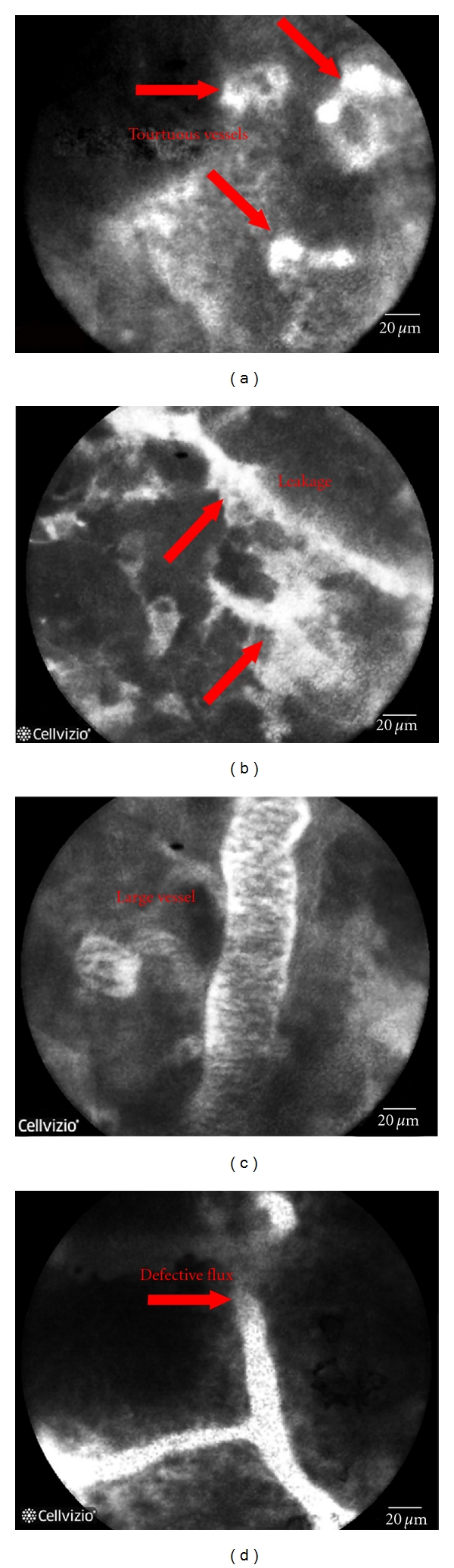
Representative pictures of the images obtained during pCLE endoscopy where the presence of tortuouse large blood vessels with defective flux and leakage are indicated by the arrows.

Endoscopic imaging and monitoring of angiogenesis have the potential to be valuable biomarkers in preneoplastic, premalignant, and cancer stage in GI lesions. The “endoscopic angiogenesis” analysis on gastric and colo-rectal cancers was performed on the patients listed in Table 1. We evaluated pCLE images from 25 sequences/biopsy sites and compared with the histological data. The vascular architecture in cancer patients was abnormal (enlarged, tortuous microvessels with altered blood flow). The morphological pattern of neoangiogenesis was in accordance with the histology and immunohistochemical analysis, allowing us to develop an arbitrary “angiogenesis” scale whose criteria are reported in Table 2. The Cannizzaro-Spessotto scale evaluates the extent of intratumoral angiogenesis based on the increase of the number of vessels, the presence of tortuous and large vessels, fluorescein leakage, and defective flux (Figure 1). Even if preliminary, these data (reported in Table 3) suggest that the application of Cannizzaro-Spessotto scale could be helpful in predicting the response to anti-angiogenic therapy and possible chemoresistance of a tumor during treatment and if the treatment received has been insufficient to avoid surgery. Further data on a greater number of tumors at different stages are needed to improve the diagnostic accuracy and to guide and predict the more appropriate individualized strategies for the treatment.

## 5. Conclusions and Perspectives

The use of pCLE allowed the *in vivo* assessment of the morphological alterations and the abnormal microvasculature of the cancer mucosa and the results correlated with the traditional conventional approach. pCLE can be considered a crucial and revolutionary method for real-time analysis of the vascularization pattern in colo-rectal and gastric cancer. These preliminary results indicate that pCLE in combination with immunological staining (CD31 and MMRN2) hold the potential for a significant impact both on basic research and clinical practice, suggesting a substantial possibility for a translational study.

##  Disclosures 

R. Cannizzaro and M. Mongiat shared first authorship.

## Figures and Tables

**Table 1:** Patients enrolled for pCLE analysis.

Patient	Age	F/M	Diagnosis	Grade/Staging (EUS)
1 DM	64	M	Adenoma	
2 ML	62	F	Rectal neoplasia	T3N+
3 GG	60	M	Gastric neoplasia	T1N0
4 CG	51	F	Rectal neoplasia	T3N+
5 ZI	71	M	Rectal neoplasia	T3M+
6 CP	63	F	Rectal neoplasia (stenosis)	
7 GM	77	F	Rectal neoplasia	T3N0
8 SS	38	M	Rectal neoplasia	T3N + M?
9 TB	68	M	Rectal neoplasia	T3Nx
10 TC	54	M	Rectal neoplasia	T3N+
11 FL	66	F	Gastric neoplasia	T3N+

**Table 2:** Calculation of Cannizzaro-Spessotto scale.

Criterion	Score	
	No	Yes
Tortuous vessels	0	1
Large vessels	0	1
Leakage	0	1
Defective flux	0	1

The patient' s angiogenesis index is obtained by summing the scores of the single items.

**Table 3:** “Angiogenetic status” of the patients.

Patient	“Angiogenesis” index (Cannizzaro-Spessotto scale)
1 DM	0
2 ML	3
3 GG	2
4 CG	2
5 ZI	Not applicable (stenosis)
6 CP	Not applicable (stenosis)
7 GM	4
8 SS	3
9 TB	2
10 TC	2
11 FL	3

## References

[1] Wallace MB, Fockens P (2009). Probe-based confocal laser endomicroscopy. *Gastroenterology*.

[2] Bajbouj M, Vieth M, Rösch T (2010). Probe-based confocal laser endomicroscopy compared with standard four-quadrant biopsy for evaluation of neoplasia in Barretts esophagus. *Endoscopy*.

[3] De Palma GD (2009). Confocal laser endomicroscopy in the “in vivo” histological diagnosis of the gastrointestinal tract. *World Journal of Gastroenterology*.

[4] De Palma GD, Staibano S, Siciliano S (2010). In vivo characterisation of superficial colorectal neoplastic lesions with high-resolution probe-based confocal laser endomicroscopy in combination with video-mosaicing: a feasibility study to enhance routine endoscopy. *Digestive and Liver Disease*.

[5] Folkman J (1971). Tumor angiogenesis: therapeutic implications. *New England Journal of Medicine*.

[6] Sökmen S, Sarioğlu S, Füzün M, Terzi C, Küpelioğlu A, Aslan B (2001). Prognostic significance of angiogenesis in rectal cancer: a morphometric investigation. *Anticancer Research*.

[7] Folkman J (1994). Angiogenesis and breast cancer. *Journal of Clinical Oncology*.

[8] Weidner N (1995). Intratumor microvessel density as a prognostic factor in cancer. *American Journal of Pathology*.

[9] Polverini PJ, Leibovich SJ (1984). Induction of neovascularization in vivo and endothelial proliferation in vitro by tumor-associated macrophages. *Laboratory Investigation*.

[10] Boehm S, Rothermundt C, Hess D, Joerger M (2010). Antiangiogenic drugs in oncology: a focus on drug safety and the elderly—a mini-review. *Gerontology*.

[11] Saad RS, Liu YL, Nathan G, Celebrezze J, Medich D, Silverman JF (2004). Endoglin (CD105) and vascular endothelial growth factor as prognostic markers in colorectal cancer. *Modern Pathology*.

[12] Brewer CA, Setterdahl JJ, Li MJ, Johnston JM, Mann JL, McAsey ME (2000). Endoglin expression as a measure of microvessel density in cervical cancer. *Obstetrics and Gynecology*.

[13] Bodey B, Bodey B, Siegel SE, Kaiser HE (1998). Over-expression of endoglin (CD105): a marker of breast carcinoma-induced neo-vascularization. *Anticancer Research*.

[14] Braghetta P, Ferrari A, De Gemmis P (2004). Overlapping, complementary and site-specific expression pattern of genes of the EMILIN/Multimerin family. *Matrix Biology*.

[15] Sanz-Moncasi MP, Garin-Chesa P, Stockert E, Jaffe EA, Old LJ, Rettig WJ (1994). Identification of a high molecular weight endothelial cell surface glycoprotein, endoGlyx-1, in normal and tumor blood vessels. *Laboratory Investigation*.

[16] Christian S, Ahorn H, Novatchkoval M (2001). Molecular Cloning and Characterization of EndoGlyx-1, an EMILIN-like Multisubunit Glycoprotein of Vascular Endothelium. *Journal of Biological Chemistry*.

[17] Huber MA, Kraut N, Schweifer N (2006). Expression of stromal cell markers in distinct compartments of human skin cancers. *Journal of Cutaneous Pathology*.

[18] Koperek O, Scheuba C, Puri C (2007). Molecular characterization of the desmoplastic tumor stroma in medullary thyroid carcinoma. *International Journal of Oncology*.

[19] Lorenzon E, Colladel R, Andreuzzi E MULTIMERIN2 impairs tumor angiogenesis and growth by interfering with VEGF-A/VEGFR2 pathway.

[20] Meining A, Wallace MB (2008). Endoscopic imaging of angiogenesis in vivo. *Gastroenterology*.

[21] Meining A, Frimberger E, Becker V (2008). Detection of cholangiocarcinoma in vivo using miniprobe-based confocal fluorescence microscopy. *Clinical Gastroenterology and Hepatology*.

[22] Becker V, Vieth M, Bajbouj M, Schmid RM, Meining A (2008). Confocal laser scanning fluorescence microscopy for in vivo determination of microvessel density in Barrett’s esophagus. *Endoscopy*.

[23] Gheonea DI, Cârţânǎ T, Ciurea T, Popescu C, Bǎdǎrǎu A, Sǎftoiu A (2011). Confocal laser endomicroscopy and immunoendoscopy for real-time assessment of vascularization in gastrointestinal malignancies. *World Journal of Gastroenterology*.

[24] Foersch S, Kiesslich R, Waldner MJ (2010). Molecular imaging of VEGF in gastrointestinal cancer in vivo using confocal laser endomicroscopy. *Gut*.

